# 2,5-Dimethyl-3-(3-methyl­thio­phen-2-yl)perhydro­pyrrolo[3,4-*d*]isoxazole-4,6-dione

**DOI:** 10.1107/S1600536808012993

**Published:** 2008-05-17

**Authors:** Mustafa Odabaşoğlu, Hamdi Özkan, Yılmaz Yıldırır, Orhan Büyükgüngör

**Affiliations:** a Department of Chemistry, Faculty of Arts & Sciences, Ondokuz Mayıs University, TR-55139 Kurupelit Samsun, Turkey; b Department of Chemistry, Faculty of Arts & Sciences, Gazi University, Ankara, Turkey; c Department of Physics, Faculty of Arts & Sciences, Ondokuz Mayıs University, TR-55139 Kurupelit Samsun, Turkey

## Abstract

The crystal structure of the title compound, C_12_H_14_N_2_O_3_S, exhibits intra­molecular C—H⋯S and inter­molecular C—H⋯S, C—H⋯O hydrogen bonds, C—S⋯N [S⋯N = 2.980 (2) Å, C—S⋯N = 145.78 (17)°] and C—H⋯π inter­actions; these inter­actions generate two *C*(5) chains and *S*(4), *S*(6) and *R*_4_^4^(28) ring motifs. The isoxazole ring has an envelope conformation; the N atom, which is the flap atom, is displaced by 0.261 (2) Å from the plane defined by the remaining four atoms. The dihedral angle between the succinimide and thio­phene rings is 46.8 (2)°.

## Related literature

For general background, see: Huisgen (1960 ▶); Black *et al.* (1975 ▶); Alibes *et al.* (2003 ▶); Kumar *et al.* (2003 ▶); Richman (2001 ▶); Chiacchio *et al.* (2003*a* ▶,*b* ▶). For ring motif details, see: Etter (1990 ▶); Bernstein *et al.* (1995 ▶). For related literature, see: Malamidou-Xenikaki *et al.* (1997 ▶); Coutouli-Argyropoulou *et al.* (1997 ▶); De Clercq (2002*a* ▶,*b* ▶,*c* ▶).
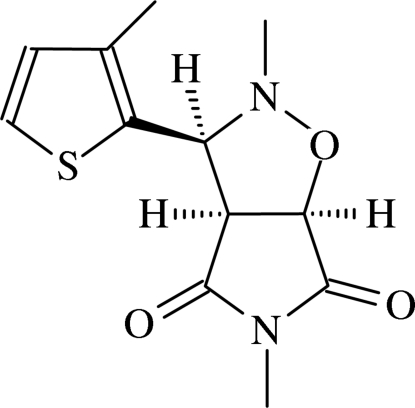


## Experimental

### 

#### Crystal data

C_12_H_14_N_2_O_3_S
*M* *_r_* = 266.31
Orthorhombic, 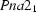

*a* = 12.0318 (10) Å
*b* = 14.6759 (9) Å
*c* = 7.2635 (4) Å
*V* = 1282.57 (15) Å^3^
*Z* = 4
Mo *K*α radiation
μ = 0.25 mm^−1^
*T* = 296 (2) K
0.52 × 0.48 × 0.43 mm

#### Data collection

STOE IPDS2 diffractometer
Absorption correction: integration (*X-RED32*; Stoe & Cie, 2002 ▶) *T*_min_ = 0.895, *T*_max_ = 0.929
12725 measured reflections
2511 independent reflections
2212 reflections with *I* > 2σ(*I*)
*R*_int_ = 0.058

#### Refinement

*R*[*F* ^2^ > 2σ(*F* ^2^)] = 0.040
*wR*(*F* ^2^) = 0.090
*S* = 1.08
2511 reflections
177 parameters
1 restraint
H atoms treated by a mixture of independent and constrained refinement
Δρ_max_ = 0.18 e Å^−3^
Δρ_min_ = −0.16 e Å^−3^
Absolute structure: Flack (1983 ▶), 1151 Friedel pairs
Flack parameter: 0.01 (9)

### 

Data collection: *X-AREA* (Stoe & Cie, 2002 ▶); cell refinement: *X-AREA*; data reduction: *X-RED32* (Stoe & Cie, 2002 ▶); program(s) used to solve structure: *SHELXS97* (Sheldrick, 2008 ▶); program(s) used to refine structure: *SHELXL97* (Sheldrick, 2008 ▶); molecular graphics: *ORTEP-3 for Windows* (Farrugia, 1997 ▶); software used to prepare material for publication: *WinGX* (Farrugia, 1999 ▶).

## Supplementary Material

Crystal structure: contains datablocks I, global. DOI: 10.1107/S1600536808012993/gw2041sup1.cif

Structure factors: contains datablocks I. DOI: 10.1107/S1600536808012993/gw2041Isup2.hkl

Additional supplementary materials:  crystallographic information; 3D view; checkCIF report

## Figures and Tables

**Table 1:** Hydrogen-bond geometry (Å, °)

*D*—H⋯*A*	*D*—H	H⋯*A*	*D*⋯*A*	*D*—H⋯*A*
C7—H7*C*⋯S1	0.96	2.97	3.467 (3)	114
C5—H5*C*⋯S1^i^	0.96	2.91	3.808 (3)	156
C8—H8⋯O3^ii^	0.88 (2)	2.56 (2)	3.426 (3)	168 (2)
C12—H12*C*⋯*Cg*1^iii^	0.96	2.94	3.693 (3)	137

Symmetry codes: (i) 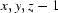; (ii) 
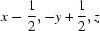
; (iii) 
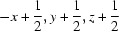
. *Cg*1 is the centroid of the S1,C1–C4 ring.

**Fig. 1.**
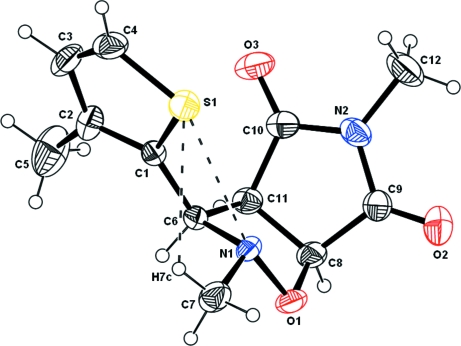
A view of (I) with the atomic numbering scheme. Displacement ellipsoids are drawn at the 30% probability level.

**Fig. 2.**
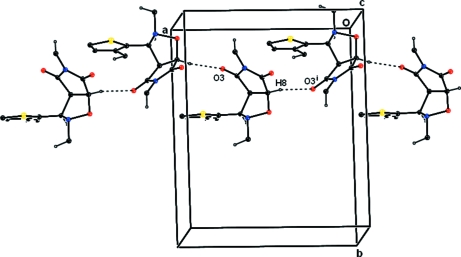
Part of the crystal structure of (I), showing the formation of C(5) chain along the [100] direction. H atoms not involved in hydrogen bonds have been omitted for clarity. The dashed line indicates a hydrogen bond. [Symmetry code: (i) 1 - x, 0.5 - y, z].

**Fig. 3.**
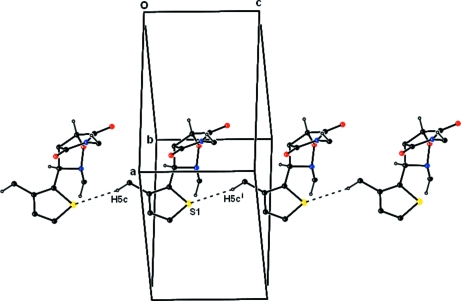
Part of the crystal structure of (I), showing the formation of C(5) chain along the [001] direction. H atoms not involved in hydrogen bonds have been omitted for clarity. The dashed line indicates a hydrogen bond. [Symmetry code: (i) x, y, z + 1].

**Fig. 4.**
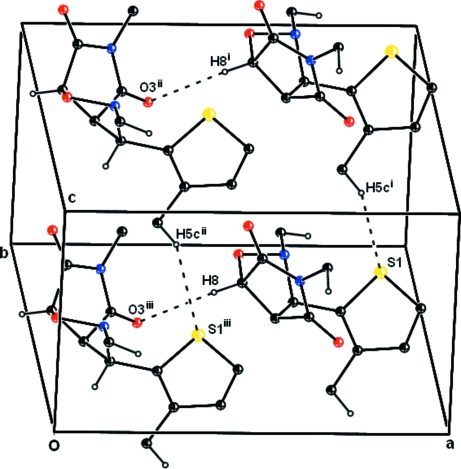
Part of the crystal structure of (I), showing the formation of R44(28) ring motif. H atoms not involved in hydrogen bonds have been omitted for clarity. The dashed line indicates hydrogen bond. [Symmetry codes: (i) x, y, z + 1; (ii) 1 - x, 1/2 - y, 1 + z; (iii) 1 - x, 1/2 - y, z].

**Fig. 5.**
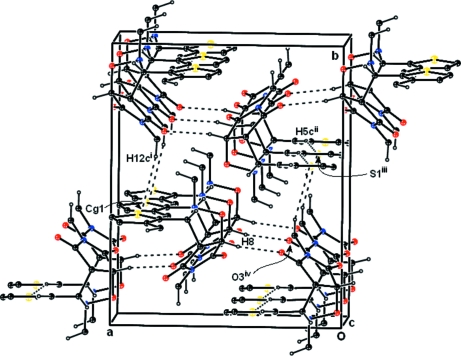
A packing diagram of (I), with hydrogen bonds drawn as dashed lines. H atoms not involved in hydrogen bonds have been omitted for clarity. [Symmetry codes: (i) 3/2 + x, 1/2 + y, 1/2 + z; (ii) 1 - x, 1 - y, -z; (iii) 1 - x, 1 - y, z - 1/2; (iv) 1/2 + x, 1/2 + y, z]

**Fig. 6.**
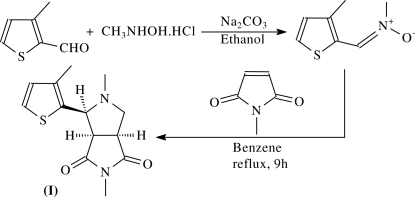
Preparation of the title compound.

## supplementary crystallographic information

### Comment

A general principle for the synthesis of five-membered rings was introduced in 1960 as 1,3-dipolar cycloaddition and turned out to be remarkably widespread (Huisgen, 1960). Because of easy 1,3-dipolar cycloaddition reactions to alkenes, alkynes, isocyanates, isothiocyanates, phospharanes, sulphenes and sulphynl compounds, nitrones are the important intermediates in synthetic organic chemistry (Black *et al.*, 1975). Especially, the nitrone-olefin 1,3-dipolar cycloaddition reaction is interesting since it can create three new contiguous stereogenic centers in a single step (Alibes *et al.*, 2003). Both inter and intramolecular nitrone cycloaddition reactions have received attention because they are useful methods for the formation of heterocycles of biological active compounds (Kumar *et al.*, 2003).

There has been an ever-increasing quest for modified nucleosides due to their potential applications in antiviral and anticancer therapies (Richman, 2001; De Clercq, 2002*a*,b,c). In a recent approach to modified nucleosides, the furanose ring has been replaced by other heterocyclic analogs (Chiacchio *et al.*, 2003*a*). Among these N and O containing five-membered heterocycles, isoxazolidines, and isoxazoline derivatives have emerged as important candidates, and have been shown to display useful anticancer and antiviral properties (Chiacchio *et al.*, 2003*b*). Consequently, synthetic studies on isoxazolidines have drawn considerable attention and 1,3-dipolar cycloadditions of nitrones afford the most straightforward route to isoxazolidines and we have described, the syntheses and crystal structure of, (I), 2,5-dimethyl-3-(3-methylthiophen-2-yl)-dihydro-2*H*-pyrrolo [3,4-*d*]isoxazole-4,6(5*H*,6aH)-dione.

The overall view and atom-labeling of the molecule of (I) are displayed in Figure 1. The hydrogen-bonding parameters are given in Table 1 and the packing arrangement of the molecules is illustrated in Figures 2–5. Compound is stabilized by intramolecular C—H···S hydrogen bond and S···N heteroatom interactions [in C1—S1···N; S···N = 2.980 (2) Å, C1—S1···N = 145.78 (17) °], which form S(4) and S(6) motifs, and intermolecular C—H···S and C—H···O hydrogen bonds and C—H···π interactions. As shown in Figures 2 and 3 the structure of the compound is made up of C8—H8···O3 and C5—H5c···S1 H-bonded polymeric bands of [C_12_H_14_N_2_O_3_S] molecules which are lined up nearly along the diagonal of the (100) (Fig. 2) and (001) (Fig. 3) planes. These polymeric C(5) chains are linked to each other and generate *R*_4_^4^(28) ring motifs (Bernstein *et al.*, 1995; Etter, 1990) (Fig. 4). The crystal packing is also stabilized by C12—H12*c*···*Cg*1 interactions (Fig. 5, Table 1). The dihedral angle between the succinimide and thiophen rings in [C_12_ H_14_ N_2_ O_3_ S] molecules is 46.8 (2) °.

### Experimental

*N*-Methyl-*C*-(-3-methylthiophen)nitrone was prepared from 3-methylthiophenecarbaldehyde, *N*-methyl-hydroxylamine hydrochloride and sodium carbonate in ethanol according to the procedure previously described (Malamidou-Xenikaki *et al.*, 1997). This substance (3 mmol, 0.465 g) and *N*-methylmaleimide (3.3 mmol, 0.370 g) was dissolved in 50 ml benzene. The reaction mixture was refluxed for 9 h monitored by TLC. After evaporation of the solvent, the reaction mixture was separated by column chromatography, using mixtures of petroleum ether and ethyl acetate (1:1) as the eluant. The *cis*-isomer, (I), was recrystallized from CHCl_3_ / n-hexane (Fig. 6) (mp: 403–405 K).

### Refinement

The aromatic and methyl H atoms included in calculated positions and refined using a riding model approximation with the constrains 0.93–0.96 Å and *U*_iso_(H) = 1.2*U*_eq_(C) for aromatic and *U*_iso_(H) = 1.0*U*_eq_(C) for methyl. The methine H atoms were found in difference Fourier map and refined freely.

### Crystal data

**Table:**

C_12_H_14_N_2_O_3_S	*F*(000) = 560
*M**_r_* = 266.31	*D*_x_ = 1.379 Mg m^−^^3^
Orthorhombic, *P**n**a*2_1_	Mo *K*α radiation, λ = 0.71073 Å
Hall symbol: P 2c -2n	Cell parameters from 12725 reflections
*a* = 12.0318 (10) Å	θ = 1.7–28.0°
*b* = 14.6759 (9) Å	µ = 0.25 mm^−^^1^
*c* = 7.2635 (4) Å	*T* = 296 K
*V* = 1282.57 (15) Å^3^	Block, colorless
*Z* = 4	0.52 × 0.48 × 0.43 mm

### Data collection

**Table:**

STOE IPDS2 diffractometer	2511 independent reflections
Radiation source: sealed X-ray tube, 12 x 0.4 mm long-fine focus	2212 reflections with *I* > 2σ(*I*)
plane graphite	*R*_int_ = 0.058
Detector resolution: 6.67 pixels mm^-1^	θ_max_ = 26.0°, θ_min_ = 2.2°
w–scan rotation method	*h* = −14→14
Absorption correction: integration (*X-RED32*; Stoe & Cie, 2002)	*k* = −18→18
*T*_min_ = 0.895, *T*_max_ = 0.929	*l* = −8→8
12725 measured reflections	

### Refinement

**Table:**

Refinement on *F*^2^	Secondary atom site location: difference Fourier map
Least-squares matrix: full	Hydrogen site location: inferred from neighbouring sites
*R*[*F*^2^ > 2σ(*F*^2^)] = 0.040	H atoms treated by a mixture of independent and constrained refinement
*wR*(*F*^2^) = 0.090	*w* = 1/[σ^2^(*F*_o_^2^) + (0.0469*P*)^2^ + 0.1351*P*] where *P* = (*F*_o_^2^ + 2*F*_c_^2^)/3
*S* = 1.08	(Δ/σ)_max_ < 0.001
2511 reflections	Δρ_max_ = 0.19 e Å^−^^3^
177 parameters	Δρ_min_ = −0.16 e Å^−^^3^
1 restraint	Absolute structure: Flack (1983), 1151 Friedel pairs
Primary atom site location: structure-invariant direct methods	Flack parameter: 0.01 (9)

### Special details

**Table:**

Geometry. All e.s.d.'s (except the e.s.d. in the dihedral angle between two l.s. planes) are estimated using the full covariance matrix. The cell e.s.d.'s are taken into account individually in the estimation of e.s.d.'s in distances, angles and torsion angles; correlations between e.s.d.'s in cell parameters are only used when they are defined by crystal symmetry. An approximate (isotropic) treatment of cell e.s.d.'s is used for estimating e.s.d.'s involving l.s. planes.
Refinement. Refinement of *F*^2^ against ALL reflections. The weighted *R*-factor *wR* and goodness of fit *S* are based on *F*^2^, conventional *R*-factors *R* are based on *F*, with *F* set to zero for negative *F*^2^. The threshold expression of *F*^2^ > σ(*F*^2^) is used only for calculating *R*-factors(gt) *etc*. and is not relevant to the choice of reflections for refinement. *R*-factors based on *F*^2^ are statistically about twice as large as those based on *F*, and *R*- factors based on ALL data will be even larger.

### Fractional atomic coordinates and isotropic or equivalent isotropic displacement parameters (Å^2^)

**Table:**

	*x*	*y*	*z*	*U*_iso_*/*U*_eq_	
C1	0.76496 (19)	0.41201 (15)	0.2036 (3)	0.0359 (5)	
C2	0.8126 (2)	0.40438 (19)	0.0347 (4)	0.0543 (7)	
C3	0.9305 (2)	0.4061 (2)	0.0477 (5)	0.0651 (9)	
H3	0.9768	0.4008	−0.0543	0.078*	
C4	0.9688 (2)	0.41604 (19)	0.2179 (5)	0.0622 (8)	
H4	1.0438	0.4198	0.2479	0.075*	
C5	0.7496 (4)	0.3958 (3)	−0.1384 (4)	0.1026 (14)	
H5A	0.6863	0.4356	−0.1349	0.103*	
H5B	0.7249	0.3340	−0.1529	0.103*	
H5C	0.7964	0.4122	−0.2401	0.103*	
C6	0.64389 (18)	0.41050 (16)	0.2458 (3)	0.0369 (5)	
C7	0.6216 (2)	0.54462 (18)	0.4463 (4)	0.0608 (7)	
H7A	0.6049	0.5621	0.5707	0.061*	
H7B	0.5670	0.5701	0.3648	0.061*	
H7C	0.6939	0.5668	0.4133	0.061*	
C8	0.5012 (2)	0.32778 (16)	0.4027 (4)	0.0477 (6)	
C9	0.5415 (2)	0.26963 (18)	0.5620 (4)	0.0523 (6)	
C10	0.6628 (2)	0.24093 (17)	0.3249 (4)	0.0482 (7)	
C11	0.5881 (2)	0.31489 (17)	0.2531 (4)	0.0441 (5)	
C12	0.6924 (3)	0.1569 (2)	0.6175 (6)	0.0829 (10)	
H12A	0.6583	0.1543	0.7369	0.083*	
H12B	0.7685	0.1756	0.6301	0.083*	
H12C	0.6895	0.0978	0.5611	0.083*	
N1	0.61980 (15)	0.44503 (14)	0.4315 (3)	0.0409 (5)	
N2	0.63330 (18)	0.22216 (14)	0.5031 (4)	0.0546 (6)	
O1	0.50134 (14)	0.42160 (11)	0.4512 (3)	0.0543 (5)	
O2	0.5015 (2)	0.26507 (16)	0.7130 (3)	0.0813 (7)	
O3	0.73562 (16)	0.20182 (14)	0.2435 (4)	0.0697 (6)	
S1	0.86334 (5)	0.42104 (5)	0.37501 (8)	0.05480 (19)	
H6	0.6036 (19)	0.4459 (14)	0.160 (3)	0.028 (5)*	
H8	0.432 (2)	0.3135 (15)	0.374 (4)	0.045 (6)*	
H11	0.561 (2)	0.2974 (15)	0.130 (4)	0.037 (6)*	

### Atomic displacement parameters (Å^2^)

**Table:**

	*U*^11^	*U*^22^	*U*^33^	*U*^12^	*U*^13^	*U*^23^
C1	0.0367 (12)	0.0340 (11)	0.0370 (11)	−0.0006 (9)	0.0001 (9)	0.0022 (9)
C2	0.0608 (16)	0.0580 (16)	0.0442 (15)	−0.0081 (13)	0.0116 (13)	−0.0019 (12)
C3	0.0514 (16)	0.0625 (18)	0.082 (2)	−0.0035 (14)	0.0339 (17)	−0.0026 (16)
C4	0.0357 (14)	0.0555 (17)	0.096 (2)	0.0034 (12)	0.0073 (14)	0.0085 (17)
C5	0.109 (3)	0.151 (4)	0.0478 (17)	−0.020 (3)	0.011 (2)	−0.020 (2)
C6	0.0326 (11)	0.0403 (12)	0.0378 (11)	0.0036 (10)	−0.0012 (10)	0.0071 (10)
C7	0.0629 (17)	0.0447 (14)	0.0747 (18)	0.0046 (13)	0.0221 (14)	−0.0023 (13)
C8	0.0284 (11)	0.0511 (15)	0.0634 (18)	−0.0013 (10)	0.0011 (12)	0.0058 (12)
C9	0.0459 (13)	0.0448 (14)	0.0664 (17)	−0.0075 (12)	0.0057 (13)	0.0077 (12)
C10	0.0356 (12)	0.0355 (13)	0.0735 (18)	−0.0046 (10)	−0.0001 (11)	−0.0017 (11)
C11	0.0344 (11)	0.0462 (14)	0.0517 (15)	−0.0007 (10)	−0.0063 (11)	−0.0006 (11)
C12	0.082 (2)	0.061 (2)	0.106 (3)	0.0089 (17)	−0.010 (2)	0.036 (2)
N1	0.0343 (10)	0.0412 (10)	0.0472 (11)	0.0010 (8)	0.0110 (8)	0.0021 (8)
N2	0.0481 (13)	0.0400 (11)	0.0758 (16)	−0.0013 (9)	−0.0049 (11)	0.0138 (11)
O1	0.0368 (9)	0.0461 (9)	0.0800 (12)	0.0083 (7)	0.0194 (8)	0.0056 (9)
O2	0.0908 (17)	0.0780 (15)	0.0750 (14)	0.0003 (13)	0.0242 (13)	0.0235 (13)
O3	0.0463 (11)	0.0520 (11)	0.1108 (16)	0.0067 (9)	0.0152 (12)	−0.0069 (11)
S1	0.0411 (3)	0.0754 (4)	0.0479 (3)	−0.0001 (3)	−0.0090 (3)	0.0068 (4)

### Geometric parameters (Å, °)

**Table:**

C1—C2	1.358 (3)	C7—H7B	0.9600
C1—C6	1.489 (3)	C7—H7C	0.9600
C1—S1	1.723 (2)	C8—O1	1.421 (3)
C2—C3	1.422 (4)	C8—C9	1.517 (4)
C2—C5	1.474 (4)	C8—C11	1.519 (4)
C3—C4	1.328 (5)	C8—H8	0.88 (3)
C3—H3	0.9300	C9—O2	1.200 (3)
C4—S1	1.708 (3)	C9—N2	1.374 (4)
C4—H4	0.9300	C10—O3	1.202 (3)
C5—H5A	0.9600	C10—N2	1.371 (4)
C5—H5B	0.9600	C10—C11	1.503 (4)
C5—H5C	0.9600	C11—H11	0.99 (3)
C6—N1	1.470 (3)	C12—N2	1.454 (4)
C6—C11	1.556 (3)	C12—H12A	0.9600
C6—H6	0.95 (2)	C12—H12B	0.9600
C7—N1	1.466 (3)	C12—H12C	0.9600
C7—H7A	0.9600	N1—O1	1.473 (2)
			
C2—C1—C6	126.7 (2)	O1—C8—C11	107.29 (19)
C2—C1—S1	111.65 (19)	C9—C8—C11	104.8 (2)
C6—C1—S1	121.65 (16)	O1—C8—H8	106.9 (15)
C1—C2—C3	111.1 (2)	C9—C8—H8	110.5 (17)
C1—C2—C5	124.1 (3)	C11—C8—H8	116.6 (19)
C3—C2—C5	124.8 (3)	O2—C9—N2	125.4 (3)
C4—C3—C2	114.2 (3)	O2—C9—C8	126.9 (3)
C4—C3—H3	122.9	N2—C9—C8	107.8 (2)
C2—C3—H3	122.9	O3—C10—N2	123.9 (3)
C3—C4—S1	111.6 (2)	O3—C10—C11	127.6 (3)
C3—C4—H4	124.2	N2—C10—C11	108.5 (2)
S1—C4—H4	124.2	C10—C11—C8	104.7 (2)
C2—C5—H5A	109.5	C10—C11—C6	113.9 (2)
C2—C5—H5B	109.5	C8—C11—C6	102.1 (2)
H5A—C5—H5B	109.5	C10—C11—H11	108.9 (14)
C2—C5—H5C	109.5	C8—C11—H11	117.1 (15)
H5A—C5—H5C	109.5	C6—C11—H11	110.3 (14)
H5B—C5—H5C	109.5	N2—C12—H12A	109.5
N1—C6—C1	112.12 (19)	N2—C12—H12B	109.5
N1—C6—C11	101.21 (19)	H12A—C12—H12B	109.5
C1—C6—C11	116.27 (19)	N2—C12—H12C	109.5
N1—C6—H6	108.4 (13)	H12A—C12—H12C	109.5
C1—C6—H6	110.9 (14)	H12B—C12—H12C	109.5
C11—C6—H6	107.2 (13)	C7—N1—C6	114.11 (19)
N1—C7—H7A	109.5	C7—N1—O1	103.87 (18)
N1—C7—H7B	109.5	C6—N1—O1	101.51 (17)
H7A—C7—H7B	109.5	C10—N2—C9	113.6 (2)
N1—C7—H7C	109.5	C10—N2—C12	123.1 (3)
H7A—C7—H7C	109.5	C9—N2—C12	123.3 (3)
H7B—C7—H7C	109.5	C8—O1—N1	101.71 (16)
O1—C8—C9	110.8 (2)	C4—S1—C1	91.40 (14)
			
C6—C1—C2—C3	−178.3 (2)	N1—C6—C11—C10	−88.1 (2)
S1—C1—C2—C3	−0.1 (3)	C1—C6—C11—C10	33.7 (3)
C6—C1—C2—C5	1.9 (5)	N1—C6—C11—C8	24.1 (2)
S1—C1—C2—C5	−179.9 (3)	C1—C6—C11—C8	145.9 (2)
C1—C2—C3—C4	−1.0 (4)	C1—C6—N1—C7	78.5 (3)
C5—C2—C3—C4	178.8 (3)	C11—C6—N1—C7	−156.9 (2)
C2—C3—C4—S1	1.5 (4)	C1—C6—N1—O1	−170.41 (17)
C2—C1—C6—N1	−164.3 (2)	C11—C6—N1—O1	−45.82 (19)
S1—C1—C6—N1	17.6 (3)	O3—C10—N2—C9	−174.5 (3)
C2—C1—C6—C11	79.9 (3)	C11—C10—N2—C9	4.2 (3)
S1—C1—C6—C11	−98.2 (2)	O3—C10—N2—C12	4.5 (4)
O1—C8—C9—O2	59.0 (4)	C11—C10—N2—C12	−176.8 (3)
C11—C8—C9—O2	174.4 (3)	O2—C9—N2—C10	−179.0 (3)
O1—C8—C9—N2	−121.1 (2)	C8—C9—N2—C10	1.0 (3)
C11—C8—C9—N2	−5.6 (3)	O2—C9—N2—C12	2.0 (5)
O3—C10—C11—C8	171.1 (2)	C8—C9—N2—C12	−177.9 (3)
N2—C10—C11—C8	−7.5 (3)	C9—C8—O1—N1	78.9 (2)
O3—C10—C11—C6	−78.2 (3)	C11—C8—O1—N1	−34.9 (2)
N2—C10—C11—C6	103.1 (2)	C7—N1—O1—C8	169.8 (2)
O1—C8—C11—C10	125.6 (2)	C6—N1—O1—C8	51.1 (2)
C9—C8—C11—C10	7.7 (3)	C3—C4—S1—C1	−1.3 (2)
O1—C8—C11—C6	6.7 (2)	C2—C1—S1—C4	0.8 (2)
C9—C8—C11—C6	−111.2 (2)	C6—C1—S1—C4	179.1 (2)

### Hydrogen-bond geometry (Å, °)

**Table:**

*D*—H···*A*	*D*—H	H···*A*	*D*···*A*	*D*—H···*A*
C7—H7C···S1	0.96	2.97	3.467 (3)	114.
C5—H5C···S1^i^	0.96	2.91	3.808 (3)	156.
C8—H8···O3^ii^	0.88 (2)	2.56 (2)	3.426 (3)	168 (2)
C12—H12C···Cg1^iii^	0.96	2.94	3.693 (3)	137.

Symmetry codes: (i) *x*, *y*, *z*−1; (ii) *x*−1/2, −*y*+1/2, *z*; (iii) −*x*+1/2, *y*+1/2, *z*+1/2.
